# Balance within the Neurexin Trans-Synaptic Connexus Stabilizes Behavioral Control

**DOI:** 10.3389/fnhum.2014.00052

**Published:** 2014-02-27

**Authors:** Raymond A. Clarke, Valsamma Eapen

**Affiliations:** ^1^Ingham Institute, School of Medicine, University of Western Sydney, Sydney, NSW, Australia; ^2^School of Psychiatry, University of New South Wales & Academic Unit of Child Psychiatry, South West Sydney (AUCS), Liverpool Hospital, Sydney, NSW, Australia

**Keywords:** neurexin, NLGN, LRRTM, CBLN, GRID, LRRN, Autism, Tourette

## Abstract

Autism spectrum disorder (ASD) is characterized by a broad spectrum of behavioral deficits of unknown etiology. ASD associated mutations implicate numerous neurological pathways including a common association with the neurexin trans-synaptic connexus (NTSC) which regulates neuronal cell-adhesion, neuronal circuitry, and neurotransmission. Comparable DNA lesions affecting the NTSC, however, associate with a diversity of behavioral deficits within and without the autism spectrum including a very strong association with Tourette syndrome. The NTSC is comprised of numerous post-synaptic ligands competing for trans-synaptic connection with one of the many different neurexin receptors yet no apparent association exists between specific NTSC molecules/complexes and specific behavioral deficits. Together these findings indicate a fundamental role for NTSC-balance in stabilizing pre-behavioral control. Further molecular and clinical characterization and stratification of ASD and TS on the basis of NTSC status will help elucidate the molecular basis of behavior – and define how the NTSC functions in combination with other molecular determinates to strengthen behavioral control and specify behavioral deficits.

## Introduction

Autism presents within a broad group of neurodevelopmental disabilities known as autism spectrum disorders (ASDs). ASDs are characterized by impaired social interaction and communication and by restricted interests and repetitive behaviors with high heritability estimates. Over 70% of individuals with autism present with intellectual disability (ID) and ~25% with epilepsy indicating overlapping etiologies in addition to secondary molecular determinates of behavior (Baird et al., 2006). There is currently no reliable biomarker, pathology, anatomical finding, or neuroimaging correlate that is specific for or predictive of ASD (Lord et al., 2000; Bauman and Kemper, 2005; Courchesne et al., 2007; Anagnostou and Taylor, 2011). Furthermore, precious little has been established regarding the precise neurological basis of ASD with many brain regions and circuits implicated (Bauman and Kemper, 2005; Courchesne et al., 2007; Amaral et al., 2008; Anagnostou and Taylor, 2011). Several competing hypotheses have been proposed to account for core behavioral deficits and ancillary symptomatic domains in ASD, but none have been widely accepted (Zoghbi, 2003; Belmonte et al., 2004; Courchesne et al., 2007; Geschwind and Levitt, 2007; Rubenstein, 2010). Genomic analyses indicate extreme genetic heterogeneity in ASD with a conservative estimation of between 380 and 820 loci implicated (Abrahams and Geschwind, 2008; O’Roak et al., 2012; Cristino et al., 2013), where many of the loci are associated with overlapping biological pathways (O’Roak et al., 2012; Cristino et al., 2013; Kenny et al., 2013; Yadav et al., 2013). Pathway overlap also extends to neuropsychiatric disorders with behavioral profiles outside the autism spectrum. In this respect, the neurexin trans-synaptic connexus (NTSC) (Clarke et al., 2012), which regulates development and maintenance of neuronal circuitry and neurotransmission is of particular relevance given its high mutation rate in ASD and other neuropsychiatric disorders (Clarke et al., 2012; O’Roak et al., 2012; Cristino et al., 2013; Kenny et al., 2013; Yadav et al., 2013).

Global genomic studies have identified numerous genes/variants and pathways implicated in the behavioral deficits associated with ASD. Cristino et al. (2013) used copy number variations (CNV) and SNP variant analyses to define 13 distinct protein modules involved in ASD including the NTSC. In addition, O’Roak et al. (2012) found high-density of mutations in the β-catenin and p53 signaling pathways consistent with the influence of both *de novo* and extremely rare inherited single nucleotide variations (SNVs) and CNVs contributing to the overall genetic risk. The wnt/β-catenin and Notch signaling pathways in neuronal development are also implicated commensurate with the importance of neuronal circuitry/boundaries and neurotransmission during development as intersecting determinates for ASD (Griswold et al., 2012; Kenny et al., 2013).

**Figure 1:**
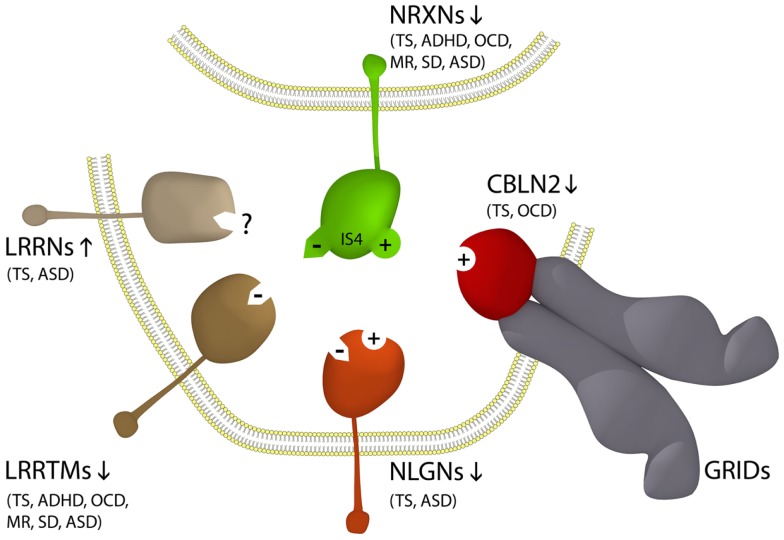
**NTSC model for ASD and Tourette Syndrome (Clarke et al., 2012) implicates the full complement of known neurexin (NRXN – green) trans-synaptic cell-adhesion ligand gene families through multiple means of enquiry including the Neuroligin (NLGN – orange); Leucine-rich repeat transmembrane protein (LRRTM – brown); Cerebellin precursor (CBLN – grey); and glutamate receptor delta (GluD/GRID – red) gene families**. The pre-synaptic NRXNs form trans-synaptic complexes with post-synaptic ligands NLGNs, LRRTMs, and CBLNs-GRIDs in the formation and/or maintenance of neuronal circuitry within the brain. Vertical arrows indicate putative pathogenic dose effects. Neurexin isoforms with (+) and without (−) the 30 amino acid insert at splice site 4 (IS4) dictate the different/competitive binding of NRXNs between ligands. Co-morbidities listed are those associated with the TS translocations and CNVs affecting the respective genes (Clarke et al., 2012).

The *neurexins* (*NRXNs*) are one of the gene families most commonly mutated in ASD (Missler et al., 2003; Belloso et al., 2007; Gauthier et al., 2011; Clarke et al., 2012; Vaags et al., 2012). NRXNs are single-pass transmembrane proteins concentrated on the pre-synaptic side of the synapse which facilitate neuronal cell-adhesion through the formation of NRXN trans-synaptic cell-adhesion complexes which together comprise the NTSC (Laurén et al., 2003; Missler et al., 2003; Chen et al., 2006; Ko et al., 2009; Linhoff et al., 2009; Wright and Washbourne, 2011; Clarke et al., 2012). The extracellular domain of pre-synaptic NRXNs binds to one of a range of post-synaptic ligands including neuroligins (NLGNs), leucine-rich repeat transmembrane proteins (LRRTMs), or cerebellin precursor (CBLN) glutamate receptor delta (Glu/GRID) complexes (Figure 1) (Missler et al., 2003; Varoqueaux et al., 2006; Ko et al., 2009; Linhoff et al., 2009; Mondin et al., 2011; Wright and Washbourne, 2011; Clarke et al., 2012; Yasumura et al., 2012). Together the three alpha-NRXNs 1–3 are essential for survival and have a pivotal role in neurodevelopment and synaptic transmission where their roles partially overlap (Missler et al., 2003) and all have been implicated in ASD (Missler et al., 2003; Belloso et al., 2007; Wang et al., 2009; Sousa et al., 2010; Gauthier et al., 2011; Clarke et al., 2012; Vaags et al., 2012; Cristino et al., 2013; Jones et al., 2013; Kenny et al., 2013; Yadav et al., 2013). However, specific NTSC components do not associate with specific behavioral deficits in ASD. Moreover, many of the same NTSC gene families are found associated with other neuropsychiatric disorders outside the autism spectrum including Tourette syndrome (TS), Asperger syndrome, schizophrenia, and ID (Clarke et al., 2012). *Neuroligin 4X* (*NLGN4X*) is just one of the many NTSC single gene overlaps between TS, ASD, and ID. The first, a *NLGN4X* truncation mutation, was identified in a family comprising two affected brothers, one with autism and ID and the other with ASD–Asperger syndrome and normal intelligence (Jamain et al., 2003). Subsequently, a different *NLGN4X* truncating mutation was identified in a multigenerational pedigree with 13 affected males with either non-syndromic ID (10 individuals), ID with ASD, or ASD without ID (Laumonnier et al., 2004). In 2008, another familial *NLGN4X* truncating mutation was identified in two brothers with TS/motor tic, one with ASD and the other with attention deficit/hyperactivity disorder (ADHD) and a mother carrier with a learning disorder, anxiety, and depression (Lawson-Yuen et al., 2008). This latter NTSC association with TS and ADHD was just the first of many such associations which have emerged since between the NTSC and the divergent behavioral profiles of ASD and TS (Clarke et al., 2012).

Tourette syndrome (TS) is characterized by motor and vocal tics, with a pre-pubertal age of onset, a waxing and waning course, and improvement in symptoms in adulthood (Eapen and Crncec, 2009). Clinical and epidemiological studies point to a very high incidence of other childhood onset behavioral and developmental disorders including up to 60% with ADHD and up to 50% with obsessive–compulsive disorder (OCD). It has long been suggested that chronic tics and OCD within TS families are likely manifestations of the same underlying genetic etiology with gender-dependent differences in expression leading to male members of the family exhibiting more tic behaviors and the female members exhibiting OCD (Eapen et al., 1993). Furthermore, recent SNP association data suggests that OCD in the presence of TS/Chronic tics may have different underlying genetic susceptibility compared to OCD alone (Eapen et al., 1993).

In the fore mentioned affected families the different behavioral profiles appear to converge around the haploinsufficiency of *NLGN4X* as the common molecular deficit. The mutation, deletion, disruption and duplication of other NTSC components are also relatively common in ASD and consistent with dose effects (Sousa et al., 2010; Gilman et al., 2011; Sakai et al., 2011; Voineagu et al., 2011; O’Roak et al., 2012; Cristino et al., 2013). Association studies also show that many of the rare variants associated with ASD occur within NTSC genes including *NRXN1-3*, *NRXN4/CNTNAP2*, *NLGN1*, *NLGN3*, *NLGN4X*, *NLGN4Y*, *LRRTM1*, *LRRTM2*, *GRID1* (Sudhof, 2008; Sousa et al., 2010; Gilman et al., 2011; Sakai et al., 2011; Voineagu et al., 2011; Clarke et al., 2012; O’Roak et al., 2012; Cristino et al., 2013) and genes encoding NTSC interacting proteins like *SHANK1-3* (Cardno and Gottesman, 2000; O’Roak et al., 2011). Moreover, recent network analyses indicate synaptic transmission as the major protein hub within the ASD network and the only protein module with interactions with all other 12 major network modules including cell–cell adhesion (Cristino et al., 2013).

The recent identification of *GRID1* associating with ASD (Cristino et al., 2013) and *CBLN1* associating with autistic-like traits (Jones et al., 2013) extends the association between the NTSC and ASD and the molecular convergence between TS and ASD. GRID1 is an inter-synaptic ligand of the post-synaptic transmembrane protein CBLN2 found associated with TS (Clarke et al., 2012), that forms the tripartite NRXN–GRID1–CBLN2 trans-synaptic cell-adhesion complex (Matsuda and Yuzaki, 2011; Clarke et al., 2012). In fact all of the gene families encoding NTSC complexes, with the exception of the *GRIDs*, have been implicated in TS including the *NRXN*, *NLGN*, *LRRTM*, and *CBLN* gene families (Petek et al., 2001; Verkerk et al., 2003; Belloso et al., 2007; Lawson-Yuen et al., 2008; Sundaram et al., 2010; Patel et al., 2011; Clarke et al., 2012; Fernandez et al., 2012). Of the 11 novel TS gene disruptions, exonic deletions, and truncations reported to date that are either recurrent or familial, a total of 9 are associated with 1 of the 20 gene families encoding the NTSC [*p* = 5.5E – 26 (*T*-test)] (Petek et al., 2001; Verkerk et al., 2003; Belloso et al., 2007; Lawson-Yuen et al., 2008; Ercan-Sencicek et al., 2010; Sundaram et al., 2010; Patel et al., 2011; Clarke et al., 2012; Fernandez et al., 2012). As such, the NTSC emerges as a primary determinate for TS (Clarke et al., 2012) and thus by inference a primary determinate for that subset of ASDs with NTSC association. Moreover, as is the case with ASD the bulk of the NTSC mutations associated with TS to date are consistent with dose effects with no apparent correlation between any of the different receptors or ligands of the NTSC and specific behavioral deficits in or between ASD and TS. Rather, the stoichiometric balance between the various competitive NTSC ligands and receptors appears to play a protective gate-keeping role in behavioral control as outlined in the pathogenetic model (Figure 1) (Clarke et al., 2012).

The striking molecular convergence between TS and ASD at the NTSC may help explain epidemiological features shared between TS and ASD but not the behavioral divergence. TS and ASD are both conditions that begin during childhood (~1% of children affected) and both are more common in males than in females. The inheritance patterns of TS are also comparable with that of ASD. TS twin studies suggest a monozygotic to dizygotic concordance of up to 77% and family studies consistently demonstrate up to a 100-fold increase in the rates of TS in first-degree relatives comparable with the high heritability of the ASDs (O’Rourke et al., 2009). ASD is also over represented in TS, and clinically, symptoms such as obsessions, compulsive behaviors, involuntary movements (tics in TS and stereotypies in ASD), poor speech control, and echolalia are common in both conditions. Furthermore, the literature suggests that around 20–40% of individuals with ASD experience tics and over 50% of individuals with ID and ASD also exhibit tics (Kadesjo and Gillberg, 2000; O’Rourke et al., 2009). Such overlap in symptoms presumably stems from the interrelated neuronal circuitry involved in the final common pathways of behavioral expression (Eapen et al., 2013). However, the divergent behaviors seen in the two conditions with motor and vocal tics in TS, and impaired social interaction and communication and restricted interests seen in ASD presumably relate to secondary/auxillary molecular and/or environmental determinants impacting neuronal circuitry development/maintenance and/or transmission.

In addition to the prevalence of NTSC dose effects in ASD and TS, the competition for connections between NRXNs and their trans-synaptic ligands (Figure 1) further supports the requirement for NTSC-balance in behavioral control. This in turn provides insight into the behavioral role of molecules linked to the NTSC. For example, the SHANK proteins which function from the post-synaptic side of the NTSC are also commonly associated with ASD. The SHANK proteins mediate attachment of the intracellular PDZ-binding domains of NTSC receptor/ligand complexes, including NRXN–NLGN and NRXN–LRRTM (Clarke et al., 2012), to the local actin-based cytoskeleton within dendritic spines. Furthermore, in Purkinje cells, the post-synaptic clustering of SHANK2 with GRID2 appears dependent on the integrity of the tripartite NRXN–GRID2–CBLN1 trans-synaptic complex (Joo et al., 2011; Matsuda and Yuzaki, 2011; Jones et al., 2013). Another TS/ASD candidate gene of related interest to the SHANKs is *synapse-associated protein 97* (*SAP97*) which encodes a scaffold-like protein located on the post-synaptic side of the synapse. Linkage analysis of a large TS pedigree identified the strongest linkage marker (D3S1311) within *SAP97* (Verkerk et al., 2006) and a male individual with TS and ASD has been identified with duplication of the *SAP97* gene locus (unpublished data), whereas micro-deletion of 3q inclusive of *SAP97* is commonly associated with schizophrenia. SAP family proteins bind directly to NTSC complexes and to NMDA, AMP, and kainate receptors at the synapse (Rumbaugh et al., 2003) and membrane-diffusing AMPARs can be rapidly trapped at SAP90/PSD95 scaffolds assembled at nascent NTSC (NRXN–NLGN) adhesions (Mondin et al., 2011). Moreover, the TS candidate ZnT3 (Clarke et al., 2012) – a synaptic zinc transporter which controls concentrations of Zn^2+^ within post-synaptic vesicles – is of particular interest here given the concentration of Zn^2+^ ions within the post-synaptic density (PSD) is known to affect the recruitment of scaffolding proteins like SHANK2 and SHANK3 (Grabrucker et al., 2011).

## NTSC Relation to Neurological Pathologies in ASD and TS

The neuronal cell-adhesion complexes of the NTSC promote synapse formation and/or maintenance bi-directionally in the glutamatergic and GABA-ergic nervous system. As such, NTSC-imbalance will translate as an imbalance in neuronal connectivity through changes in synapse patterning and transmission (Missler et al., 2003; Varoqueaux et al., 2006; Ko et al., 2009; Linhoff et al., 2009; Clarke et al., 2012). Loss of CBLN2, as reported in TS (Clarke et al., 2012), is associated with reduced mediation of inhibitory synaptogenesis (Yasumura et al., 2012). This however, appears in opposition with the reduced number of excitatory synapses associated with the downregulation of NLGN4X or the LRRTMs in TS (Figure 1) (Ko et al., 2009; Wright and Washbourne, 2011), albeit the recurrent loss/disruption of NRXN1 in TS and ASD infers loss of both excitatory and inhibitory synaptic connections. Together these findings further reinforce the importance of a balanced NTSC repertoire rather than “specific complexes” as the basis of NTSC related behavioral disorders.

Synaptic homeostasis depends on the balance between the strength of excitation, inhibition, and the intrinsic excitability of the neuronal circuitry. Evidence suggests that the balance between excitation and inhibition is tightly regulated with even small changes affecting neuronal firing (Atallah and Scanziani, 2009; Pouille et al., 2009). When this balance is perturbed, mechanisms come into play to restore synaptic homeostasis by modifying the balance between excitatory and inhibitory inputs or the application of intrinsic mechanisms to modify the balance of inward and outward voltage-dependent current (Gainey et al., 2009). Synapses are formed even when αNRXN l is deleted from the mouse genome, however, this compromises synaptic transmission (Missler et al., 2003). The pre-synaptic co-assembly of Ca^2+^ channels with the secretory apparatus is a prerequisite for the release of neurotransmitters like glutamate and this channel function is impaired in αNRXN1 knockout mice with consequent reductions in neurotransmitter release (Missler et al., 2003). The NTSC trans-synaptic connections NRXN–NLGN and NRXN–LRRTM are both sensitive to extracellular Ca^2+^ concentrations which appear to trigger post-synaptic differentiation and control the balance of inhibitory GABA-ergic and excitatory glutamatergic inputs. Glutamate, the main excitatory neurotransmitter in the vertebrate brain, has a major role in cortico-striatal-thalamo-cortical circuits and several lines of evidence support the role of glutamate in TS including: the TS association of glutamate receptors that are localized in the cellular membranes of both neurons and glia; the recognized extensive interaction between glutamate and dopamine systems; results of familial genetic studies; and data from neurochemical analyses of post-mortem brain samples (Felling and Singer, 2011; Clarke et al., 2012). Interestingly, LRRTM1 null mice have altered distribution of the excitatory pre-synaptic vesicular glutamate transporter VGLUT1 (Ko et al., 2009; Linhoff et al., 2009). Furthermore, loss of excitatory synaptic connections results in a hypo-glutamatergic state that is consistent with a loss in the synaptic weight, which is an all important factor for the circuit strength required in language development (Matsuda and Yuzaki, 2011).

## Neural Circuitry as a Function of Synaptic Pruning and Boundary Formation

Synaptic pruning plays an important role during maturation of the brain by limiting neural circuitry, and neural circuitry within specific brain regions is implicated in behavioral control. As such the integrity of neural/brain boundaries may be a factor in neuropsychiatric disorders. In this respect, it is most interesting to note that both ASD and TS have been associated with leucine-rich repeat neuronal (LRRN) type I transmembrane protein genes. *LRRN3* is localized within the genomic region most commonly duplicated in ASD (Kroisel et al., 2001; Maestrini et al., 2010; Pagnamenta et al., 2010). *LRRN3* is also nested in an antisense orientation within the *IMMP2L* gene recurrently disrupted in TS and ASD (Clarke et al., 2012). Moreover, the nearest gene relation to *LRRN3*, *LRRN1*, has been duplicated in ASD (Davis et al., 2009). These associations suggest increased dose of LRRN1 and LRRN3 maybe pathogenic for ASD and TS. Little is known about the function of LRRN3, however, LRRN1 is known to have a key role in regional boundary formation within the brain (Chen et al., 2006; Tossell et al., 2011). Studies in the developing chick demonstrate that the midbrain–hindbrain boundary (MHB) is established through the down regulation of Lrrn1 by Fgf8 on the posterior side of the future boundary (Tossell et al., 2011), thereby creating a differential cellular affinity between the two compartments likely to involve an as yet unspecified extracellular binding partner for Lrrn1. Lrrn1 in turn regulates the expression of the *Lunatic Fringe* gene which modulates Notch signaling to complete MHB formation. Over-expression of Lrrn1 disrupts the MHB with mixing of cells between compartments (Tossell et al., 2011). For further insight into this association see (Clarke et al., 2012).

## Auxillary Molecular and Environmental Determinants Specify Behavioral Deficits

Imbalance in the NTSC appears to be sufficient for but not definitive in specifying the nature of behavioral pathogenesis. Moreover, recent evidence suggests that numerous gene variants combine with environmental and physiological factors to specify behavioral deficits. For example, the sex-specific imprinting of *NRXN4/CNTNAP2*, *CTNNA3*, and *LRRTM1* is known to alter the expression of these genes and their parent-of-origin phenotypic inheritance patterns (Oudejans et al., 2004; Francks et al., 2007). Thus, a particular phenotypic co-morbidity may present based on the type and level of involvement of the different NTSC neurotransmitter pathways in combination with secondary determinates that mediate or modulate NTSC pathways during neurodevelopment whereas an early environmental insult could specify an alternate behavioral deficit/neural outcome (Herbert, 2010) including effects associated with prematurity, perinatal trauma, hypoxia, injury oxidative stress, inflammations, infections and autoimmunity, neural and psychosocial stressors, gender effects, etc. (Eapen, 2011). Gender-specific differences exist in the topographic segregation and functionality of GABA-A systems in the substantia nigra, moreover, circulating testosterone is essential for the development of the substantia nigra region in the neonatal period and to a lesser extent for final maturation in the peripubertal period (Veliskova and Moshe, 2001). In this regard, a role for testosterone has been suggested in the extreme male brain hypothesis in ASD (Baron-Cohen, 2002). Similar mechanisms may affect the TS genes leading to gender-dependent difference in phenotypic expression – with male members of TS families exhibiting more tic behaviors and female members more OCD (Eapen et al., 1993, 1997). Thus, an NTSC related imbalance that impacts development of different neuronal regions and circuitry maybe further specified by secondary genetic and/or environmental events (Eapen et al., 2014). The penetrance of the different co-morbidities may also be related to gender, gene dose effects, or the timing of events when different brain regions are being formed, thus resulting in different clinical phenotypes (Eapen et al., 2013).

## Conclusion

The NTSC provides an invaluable window into the molecular basis of behavior. The role of NTSC-balance as a gate keeper of behavioral control provides a firm basis for more in depth molecular and clinical characterization and stratification of behavioral disorders. To this end, NTSC’s common association with ASD and Tourette syndrome provides the ideal starting point for molecular and clinical comparisons between select ASD and TS families.

## Conflict of Interest Statement

The authors declare that the research was conducted in the absence of any commercial or financial relationships that could be construed as a potential conflict of interest.
